# Plasmid DNA Prime/Protein Boost Vaccination against *Campylobacter jejuni* in Broilers: Impact of Vaccine Candidates on Immune Responses and Gut Microbiota

**DOI:** 10.3390/pharmaceutics15051397

**Published:** 2023-05-03

**Authors:** Noémie Gloanec, Muriel Guyard-Nicodème, Raphaël Brunetti, Ségolène Quesne, Alassane Keita, Marianne Chemaly, Daniel Dory

**Affiliations:** 1 GVB—Viral Genetics and Biosafety Unit, French Agency for Food, Environmental and Occupational Health & Safety (ANSES), 22440 Ploufragan, France; 2 HQPAP—Unit of Hygiene and Quality of Poultry and Pork Products, French Agency for Food, Environmental and Occupational Health & Safety (ANSES), 22440 Ploufragan, France; 3 UFR of Life Sciences Environment, University of Rennes 1, 35700 Rennes, France; 4 SELEAC—Avian Breeding and Experimental Department, French Agency for Food, Environmental and Occupational Health & Safety (ANSES), 22440 Ploufragan, France

**Keywords:** systemic and mucosal immune response, antibodies, cytokines, β-defensins, *Campylobacter jejuni* caecal colonisation, caecal microbiota composition

## Abstract

*Campylobacter* infections, traced to poultry products, are major bacterial foodborne zoonoses, and vaccination is a potential solution to reduce these infections. In a previous experimental trial using a plasmid DNA prime/recombinant protein boost vaccine regimen, two vaccine candidates (YP437 and YP9817) induced a partially protective immune response against *Campylobacter* in broilers, and an impact of the protein batch on vaccine efficacy was suspected. This new study was designed to evaluate different batches of the previously studied recombinant proteins (called YP437A, YP437P and YP9817P) and to enhance the immune responses and gut microbiota studies after a *C. jejuni* challenge. Throughout the 42-day trial in broilers, caecal *Campylobacter* load, specific antibodies in serum and bile, the relative expression of cytokines and β-defensins, and caecal microbiota were assessed. Despite there being no significant reduction in *Campylobacter* in the caecum of vaccinated groups, specific antibodies were detected in serum and bile, particularly for YP437A and YP9817P, whereas the production of cytokines and β-defensins was not significant. The immune responses differed according to the batch. A slight change in microbiota was demonstrated in response to vaccination against *Campylobacter*. The vaccine composition and/or regimen must be further optimised.

## 1. Introduction

*Campylobacter* infections are the major bacterial foodborne zoonoses in Europe, with 127,840 human cases in 2021 [1]. Campylobacteriosis also affects developing countries, where the need for control strategies against this disease is growing [2]. The major sources of infections in humans are contaminated poultry products, but not only those; *Campylobacter* was also isolated from other animal species such as sheep, goats, pigs, cattle, monkeys, horses, seals, cats, and dogs, as well as from insects [3,4,5]. These infections are most often characterised by gastroenteritis, but *Campylobacter* may also be associated with a severe demyelinating neuropathy known as Guillain–Barré syndrome [6]. *Campylobacter jejuni*, a gram-negative and microaerophilic bacterium, is the most frequently reported species, causing approximately 88% of cases [1].

In poultry, the major site of colonisation is the caecum, which can contain large quantities of *C. jejuni* (about 8 log_10_ CFU/g [7,8,9], but possibly up to approximately 11 log_10_ CFU/g [10,11]). A positive correlation is usually found between the counts of *Campylobacter* in the caeca of broilers and the counts of these bacteria on chicken carcasses after slaughter [8]. In Europe, the prevalence of *Campylobacter* in broiler batches and carcasses was estimated at 71.2% and 75.8%, respectively [12].

The high levels of avian intestinal *Campylobacter* loads impact the incidence of human campylobacteriosis. In fact, it was estimated that a 2 log_10_ to 3 log_10_ CFU/g reduction of these loads in broiler caeca may reduce the relative risk of these human diseases from 42% to 58% [13].

Vaccination could be one of the control strategies at the primary poultry level to reduce these infections. Multiple vaccine development strategies targeting *Campylobacter* have been investigated in poultry. Most include subunit vaccine delivery, whether with a vector or not, and whole-cell inactivated vaccines. Moreover, different routes of administration (intramuscular, oral, and in ovo) have been tested. However, these studies have produced inconsistent results on intestinal *Campylobacter* loads in poultry [14,15,16,17,18,19,20,21,22,23,24,25,26,27,28,29,30,31]; consequently, no effective vaccine is available to date.

Interactions between the chicken gut microbiota and immune system in response to *Campylobacter* colonisation were reported in recent studies [32,33,34,35]. More specifically, *Campylobacter* was recently associated with the production of β-defensins, which are host defence peptides (HDPs), and cytokines more specifically belonging to the Th17 pathway [32,36,37]. The results concerning microbiota composition have also proved inconsistent, though *Campylobacter* colonisation has mainly been associated with an increase in *Firmicutes*. Furthermore, recent studies have evaluated the impact of vaccination against *Campylobacter* on the chicken immune system and gut microbiota [15,17,18].

A previous study by our group identified, through reverse vaccinology from the *Campylobacter* genome, two proteins—WP_002869420.1, called here YP437 (a haemolysin secreting/activating protein of the ShlB/FhaC/HecB family), and WP_002868767, called here YP9817 (hypothetical protein)—as potential vaccine candidates [38]. In an initial in vivo trial using a plasmid DNA prime/recombinant protein boost vaccine regimen, these vaccine candidates induced a partial protective immunity in broilers, reflected through the production of antibodies against *Campylobacter* and significantly lower *Campylobacter* contamination in the vaccinated groups than in the placebo group. However, in a second trial, this reduction in *Campylobacter* colonisation was not confirmed, despite the production of antibodies. During this second trial, a high inter-individual variability of colonisation (including in the placebo group) was observed using the same batch of the YP437 protein [24]. In addition, another batch of YP9817 vaccine was used, suggesting that protein batches could have impacted vaccine efficacy for this antigen (for example, due to a different protein structure or purity) [24]. Therefore, these vaccine candidates required a reassessment.

The objectives here were to: (1) re-evaluate the protective potential of the vaccine candidates against *Campylobacter* colonisation using the DNA prime/protein boost protocol with new batches of recombinant proteins (YP437P and YP9817P) and a previous one (YP437A); (2) evaluate the impact of protein batch production on vaccine efficacy with the YP437A and YP437P batches; (3) study in greater depth the innate or specific immune responses generated and changes in gut microbiota after vaccination in order to potentially identify key parameters involved in protection.

## 2. Materials and Methods

### 2.1. DNA Vaccines Production

pcDNA3-based plasmids, encoding *C. jejuni* antigens (YP437 or YP9817) or not, were produced as described previously [24]. Briefly, transformed *E. coli* Top10 with pcDNA3-YP437, pcDNA3-YP9817, or pcDNA3 were cultured in LB broth + Amp. Thereafter, the plasmids were extracted and purified using the NucleoBond PC 10000 Endotoxin Free extraction kit (ThermoFisher Scientific, Villebon sur Yvette, France) according to the manufacturer’s instructions. Plasmid concentrations were assessed by measuring the optical density at 260 nm.

For each chicken, 150 µg of plasmid DNA was mixed with 25 µg of a phosphorothioate-based backbone unmethylated CpG ODN2007 (5′-TCGTCGTTGTCGTTTTGTCGTT-3′) (Sigma-Aldrich, Saint-Quentin-Fallavier, France) and then stored at −20 °C until vaccination.

### 2.2. Production of the Recombinant Protein Vaccines

Recombinant YP437A was of the same batch as the one used previously [24], whereas new batches of recombinant YP437P and YP9817P were produced by ProteoGenix (Schiltigheim, France). For each chicken, 100 µg of recombinant protein (or phosphate buffer saline (PBS) for the placebo group) was emulsified in MONTANIDE™ ISA 78 VG (37/63, *w*/*w*) (Seppic, La Garenne-Colombes, France) using an Ultra Turrax Tube drive (IKA^®^-Werke GmbH, Staufen, Germany) the day before vaccination according to the manufacturer’s recommendations. It was then stored at 4 °C.

### 2.3. Campylobacter Strain and Growth

The *C. jejuni* C97Anses640 strain, isolated at the ANSES laboratory in Ploufragan (France) from broilers and belonging to the ST−45 (sequence type) complex, was orally delivered to the chicken as a challenge experimental infection, as described previously [17]. Briefly, frozen bacteria were plated at 41.5 °C under microaerobic conditions (85% N_2_, 10% CO_2_ and 5% O_2_) twice on selective modified charcoal cefoperazone deoxycholate agar (mCCDA) (Thermo Fisher Diagnostics, Dardilly, France) for 48 h, then inoculated twice in Brucella broth (Becton Dickinson, Le Pont-de-Claix, France) for 24 h. The bacterial suspension was diluted to 5 log_10_ CFU/mL in tryptone salt broth (BioMérieux, Bruz, France) and titrated on mCCDA plates in 10-fold dilution series.

### 2.4. Avian Vaccine Experiment

**Figure 1:**
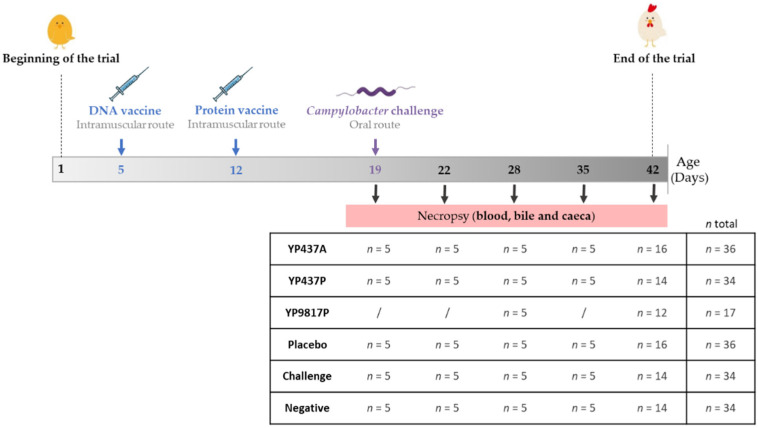
The experimental design. The number of chickens (*n*) necropsied per group on each euthanisation day is shown. The YP9817P group was composed of fewer chickens, because a smaller quantity of recombinant proteins was available than for the other groups (YP437A and YP437P) after purification.

The trial was carried out at the Animal Biosafety Level 2 facilities of ANSES’s Ploufragan Laboratory (France), an approved establishment for animal experimentation (No. E-22-745-1). It was authorised by the French Ministry of Higher Education, Research and Innovation (reference APAFIS#27000-2020082011122238 v2). A total of 191 day-of-hatch conventional Ross 308 broiler chicks (male and female) were purchased from a local hatchery. Before the trial, the absence of *Campylobacter* spp. was confirmed in husbandries (including the feeding and drinking systems) and in four chicks according to standard NF EN ISO 10272-1 (2017). The remaining chickens were randomly divided (from 17 to 36 chickens, Figure 1) into different groups (YP437A, YP437P, YP9817P, placebo, challenge and negative) and were kept in 3.42 m^2^ floor pens (1.85 × 1.85 m^2^). The YP9817P group was composed of fewer chickens because a smaller quantity of protein was available than for the other groups (YP437A and YP437P) after purification. Litter was composed of unused wood shavings. From day 1 to day 7, there were 23 h of lighting per day. Thereafter, the number of lighting hours per day was reduced to eighteen. The ambient temperature was gradually reduced from 32 °C on day 1 to 18 °C on day 35. The poultry diets were obtained from a commercial feed meal company. All the groups received a starter-grower diet from days 1 to 22, then a grower-finisher diet until day 42 (D42).

The chickens were intramuscularly injected, using 26 G needles with DNA vaccine on day 5 and protein vaccine on day 12. On D19, all the chickens except those in the negative group were orally challenged with 10^4^ CFU of *C. jejuni* C97Anses640 [17,24]. At several time-points from D19 to D42, caeca, blood and bile were sampled and processed from 5 to 16 euthanised animals per group, as previously described [17], through to determination of the systemic humoral immune response, the mucosal immune response, and *Campylobacter* spp. enumeration and microbiota analysis, respectively. Each bird was individually weighed on days 5, 12, 19, and on each slaughter day. They were observed daily to detect any occurrence of adverse reactions. The experimental design is summarised in Figure 1.

### 2.5. Campylobacter Caecal Enumeration

*Campylobacter* enumerations were assessed in caecal contents as described previously [17]. Briefly, homogenised caeca were serially diluted 1:10 (*w*/*v*) up to dilution 10^−6^ in tryptone salt broth (Biomerieux, Craponne, France) and plated on mCCDA using easySpiral (Interscience, Saint-Nom-la-Bretèche, France), an automatic plater. After incubation of the plates for 48 h at 41.5 °C under microaerobic conditions, typical *Campylobacter* colonies were enumerated and converted to log_10_ CFU/g. The detection limit for enumeration of *Campylobacter* was 2 log_10_ CFU/g of caecal content. One mL of the 10^−1^ dilution of caecal content was centrifuged (10,000× *g*, 10 min) and the pellet was stored at −70 °C until microbiota analysis.

### 2.6. Specific Serum (IgY) and Bile (IgA) Antibodies Levels against Vaccine Antigens Determined by ELISAs

The levels of specific antibodies against YP437A, YP437P, and YP9817P proteins in vaccinated groups and control groups in serum and bile were measured by ELISA as in the previous study [17], but the technic was adapted to each vaccine antigen. Briefly, 96-well Maxisorp^®^ plates coated with 100 µL of individual purified protein per well were successively incubated with diluted serum or diluted bile and diluted goat anti-chicken IgY-horseradish peroxidase (HRP) antibodies (Abcam, Paris, France), or diluted goat anti-chicken IgA-HRP (Abcam, Paris, France), respectively (according to the concentrations or dilutions indicated in Table 1). After incubation with the HRP substrate (*o*-phenylenediamine dihydrochloride in citrate buffer containing hydrogen peroxide), 1 M of H_2_SO_4_ was added. The optical densities were then measured at 490 nm using the Infinite 200 PRO Nanoquant (Tecan, Lyon, France). Each sample was measured in duplicate.

### 2.7. Total RNA Extraction and Relative Cytokine and β-Defensin Expressions Determined by Relative RT-qPCR

Total RNA was extracted from caecal tissues using an Agencourt^®^ RNAdvance™ Tissue kit (Beckman coulter, Brea, CA, USA) with magnetic beads according to the manufacturer’s protocol with modifications, as described previously [17]. Then, the eluted RNA was processed with the Turbo DNA-free™ kit (Thermo Fisher Scientific, Vilnius, Lithuania) and quantified using the Qubit RNA high sensitivity assay kit (Life Technologies, Saint-Aubin, France) with a Qubit 2.0 fluorometer (Life Technologies, Saint-Aubin, France). Some RNA samples were checked for quality and integrity by capillary electrophoresis using a fragment analyser system (Agilent, Les Ulis, France).

Total RNA (320 ng) was reverse-transcribed into cDNA using the High-capacity cDNA Reverse Transcription kit (Thermo Fisher Scientific, Villebon sur Yvette, France) according to the manufacturer’s instructions.

Quantitative PCR (qPCR) reactions were performed on a cDNA obtained from 4 ng of total RNA in duplicate using the SYBR Green Master mix (Thermo Fisher Scientific, Villebon sur Yvette, France) and the 7500 real-time PCR system (Applied Biosystems, Villebon sur Yvette, France) as described previously [17]. The primers used for β-actin, interferon (IFN)-γ, interleukin (IL)8 like (L)1, IL8L2, IL-1β, IL-4, IL-10, IL-17A, host defence peptide avian β-defensins 10 (AvBD10), and AvBD12 amplifications have been previously described [17], and the primers for IL-6 and glyceraldehyde 3-phosphate dehydrogenase (GAPDH) are presented in Table 2. Specific primers for GAPDH were designed using Primer Express^®^ (Thermo Fisher Scientific, Villebon sur Yvette, France) software. qPCR on the total RNA was performed to check that there was no residual genomic DNA in each sample. PCR efficiency, evaluated using the standard curve slope, ranged from 90 to 110%. The results were normalised with the reference genes β-actin and GAPDH as already used [39,40]. Statistical tests were performed using the mean of duplicates of 2^−ΔCt^ data for each gene between the two reference genes [17,32,41]. The gene expression, represented by delta Ct, corresponds to the average of the differences between the duplicate Ct for each gene and the duplicate Ct for each of the two reference genes (GAPDH and β-actin).

### 2.8. Statistical Analyses

R software (version 4.0.3) was used for statistical analysis [43]. To compare vaccinated groups and control groups, an ANOVA parametric test was used when the normality and homogeneity criteria of the variances had been previously validated (checked by the Shapiro–Wilk normality test and Bartlett’s test, respectively) followed by the Tukey test; otherwise, the non-parametric Kruskall-Wallis test was used followed by the Wilcoxon test. A *p*-value lower or equal to 0.05 (*p* ≤ 0.05) was considered statistically significant.

### 2.9. Caecal Microbiota Diversity Analysis

#### 2.9.1. Bacterial DNA Extraction

Bacterial DNA was isolated from caecal pellets prepared in part 2.5 using the NucleoMag Tissue Kit (Macherey-Nagel, Hoerdt, France), as described previously [17], and using caecal samples randomly chosen from the different groups. Briefly, these pellets were resuspended in 500 µL of T1 lysis buffer and mechanically homogenized by adding one stainless-steel 5 mm bead (Qiagen, Courtaboeuf, France), using the Star Beater (3 min–30 Hz) (VWR, Fontenay-sous-Bois, France). Proteinase K (Macherey Nagel, Hoerdt, France) was added, and the suspension was heated 30 min at 70 °C. After centrifugation (2 min at 13,000× *g*), DNA was extracted into standard 96-well plates from 225 µL of supernatant using the KingFisher Duo Prime instrument (Thermo Fisher Scientific, Villebon sur Yvette, France). A negative extraction control was included in each plate. DNA concentrations were estimated using the Qubit dsDNA High Sensitivity Assay Kit (Thermo Fisher Scientific, Illkirch-Graffenstaden, France) and a Qubit 2.0 Fluorometer (Life Technologies, Saint-Aubin, France). The DNA extracts were stored at −20 °C until sequencing.

#### 2.9.2. V3/V4 Variable Region of the 16S Ribosomal Genes Sequencing

Bacterial genomic DNA was PCR-amplified using a primer set covering the V3-V4 variable regions of the 16S rDNA gene (forward primer: 5′-TCGTCGGCAGCGTCAGATGTGTATAAGAGACAG-3′; reverse primer: 5′-GTCTCGTGGGCTCGGAGATGTGTATAAGAGACAG-3′) to obtain an amplicon size of about 460 bp (15044223 Rev B adapted). Amplicons were sequenced using the 2 × 300 bp paired-end sequencing strategy, which was using an Illumina MiSeq sequencer with the Illumina MiSeq reagent kit 600 version 3, according to the Illumina 16S metagenomic library preparation protocol (15044223 Rev B adapted) and the MiSeq system denature and dilute libraries guide (15039740–3 December 2017 adapted).

#### 2.9.3. Sequence Analyses

Sequences were processed using FROGS (Version 4.0.1) [44], a galaxy-supported pipeline. Briefly, paired-end reads were merged using VSEARCH, and sequences with ambiguous bases, unexpected lengths (<380 or >500 nucleotides) or with at least one primer sequence at 3′- and/or 5′-ends that did not completely correspond to the original primer sequences were removed before dereplication. The sequences were next clustered into operational taxonomic units (OTUs) using SWARM, and following FROGS guidelines, chimeras were removed using VSEARCH combined with a cross-sample validation step. OTUs with an abundance below 5 × 10^−5^ and present in fewer than 12 samples were removed, and the BLAST algorithm was used for taxonomic assignment against the SILVA 16S database (version 132 filtered at a pintail score of 80).

#### 2.9.4. Statistical Analyses of the Diversity and Structure of Caecal Microbiota

Diversity and structure of caecal microbiota statistical analyses were performed using the phyloseq R package implemented in FROGS. The richness represents the number of OTUs present in a sample (observed richness). Total richness is estimated by the Chao 1 richness index (observed richness and not observed unknown number of species present in the community). The Shannon and InvSimpson indices were used to describe diversity in samples and to take into account richness and evenness (Table S1). The effect of the vaccination and *Campylobacter* colonisation on these indices was investigated using an ANOVA followed by the Tukey test. The impact of vaccination on microbiota diversity and structure was also investigated. A weighted UniFrac (wUniFrac) distance matrix, taking into account the relative abundance of OTUs shared between samples, was calculated after data rarefaction and plotted using multidimensional scaling (MDS) to investigate the structure of the bacterial community. An ADONIS pairwise test was used to check significance. The groups’ relative abundance of the major phyla and nine main genera was compared with the non-parametric Kruskall-Wallis test. Significant differences (*p* ≤ 0.05) between groups were estimated using the Wilcoxon test. To identify and visualise whether taxa with differential abundance (Table S2) were statistically different between groups, the linear discriminant analysis (LDA) effect size (LEfSe) method was applied with an LDA > 2.

## 3. Results

### 3.1. Clinical Observations and Body Weight

No adverse reactions were observed after each vaccination or inoculation. Results showed that chicken growth was unaffected by vaccinations (including the placebo) and/or the challenge, as there was no difference in mean body weight between each group tested from D5 to D42 compared with the negative group (*p* > 0.05) (except for the placebo group only on D19) (Table 3).

### 3.2. Campylobacter Counts in the Caeca of the Infected Chickens

**Figure 2:**
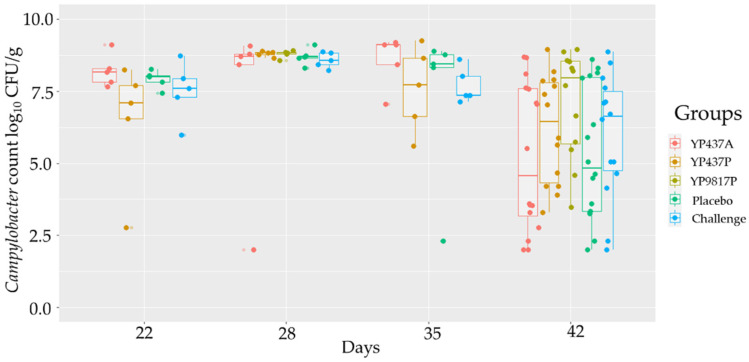
Caecal *Campylobacter* load from D22 to D42 in vaccinated groups and control groups. Each point represents one chicken. For each time point, the inter-group caecal *Campylobacter* load was compared using an ANOVA parametric test when the normality and homogeneity criteria of the variances had been validated; otherwise, the non-parametric Kruskall-Wallis test was applied. Significant differences (*p* ≤ 0.05) between groups were estimated using the Tukey test after an ANOVA parametric test or the Wilcoxon test after the Kruskall-Wallis test.

*Campylobacter* levels were measured in caecal contents at several time points after the experimental challenge on D19. Three days post-challenge, chickens from all the groups were found to be highly colonised by *Campylobacter* (mean colonisation from 2.77 to 9.11 log_10_ CFU/g). The YP9817P group was checked only on D28 and had the same level of colonisation as the other groups (mean colonisation from 2.00 to 9.11 log_10_ CFU/g) (Figure 2). No significant difference (*p* > 0.05) was observed in caecal *Campylobacter* load among the groups from D22 to D42 (Figure 2). However, a high inter-individual variability was observed on D42 in all the tested groups, with *Campylobacter* loads ranging from 2.00 to 8.94 log_10_ CFU/g (Figure 2). As expected, *Campylobacter* was not detected in the negative group (limit of detection: 2 log_10_ CFU/g).

### 3.3. Specific Serum (IgY) and Bile (IgA) Anti-YP Antibody Levels Assessed by Specific ELISAs

ELISAs were performed to determine the levels of specific systemic antibodies in serum (IgY) and specific mucosal antibodies in bile (IgA) against the three proteins tested.

**Figure 3:**
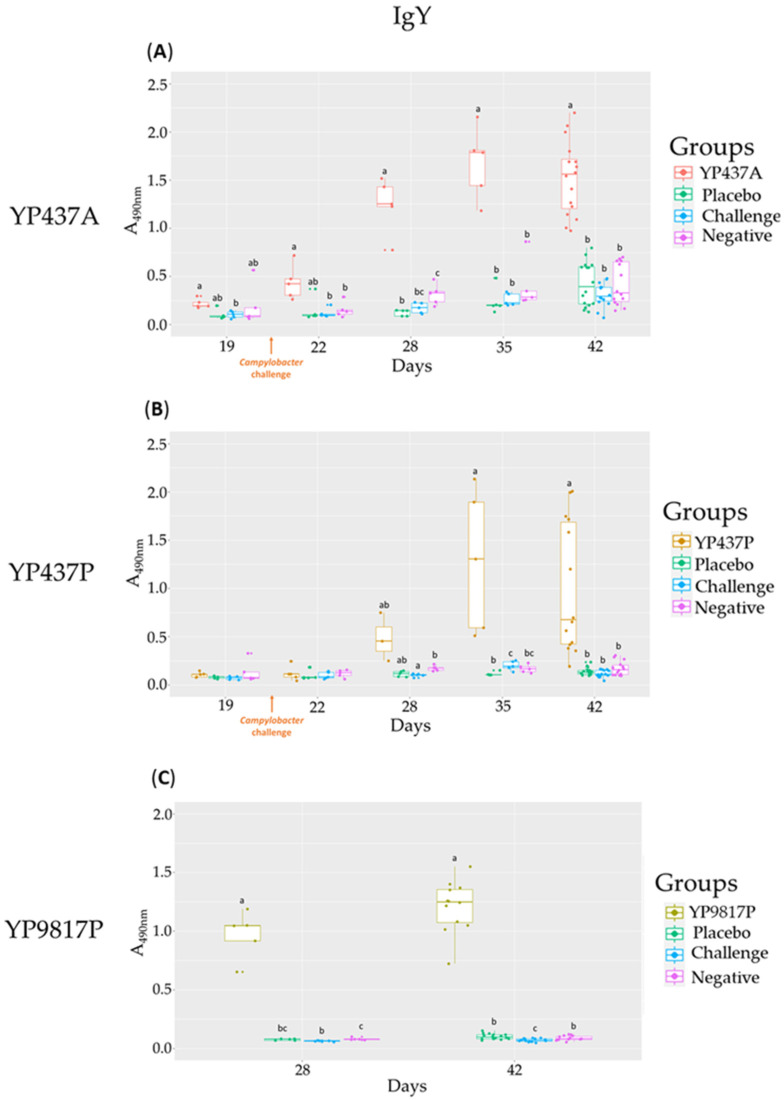
Specific anti-YP437A, anti-YP437P and anti-YP9817P IgY levels in serum from D19 to D42 in vaccinated groups and control groups. Each point represents one chicken. (**A**) Specific anti-YP437A IgY (**B**) Specific anti-YP437P IgY (**C**) Specific anti-YP9817P IgY. To compare antibody levels between the groups on each test day, an ANOVA parametric test was used when the normality and homogeneity criteria of the variances had been validated; otherwise, the non-parametric Kruskall-Wallis test was applied. Significant differences (*p* ≤ 0.05) between groups estimated using the Tukey test after an ANOVA parametric test or the Wilcoxon test after the Kruskall-Wallis test are represented by superscript letters ^abc^.

**Figure 4:**
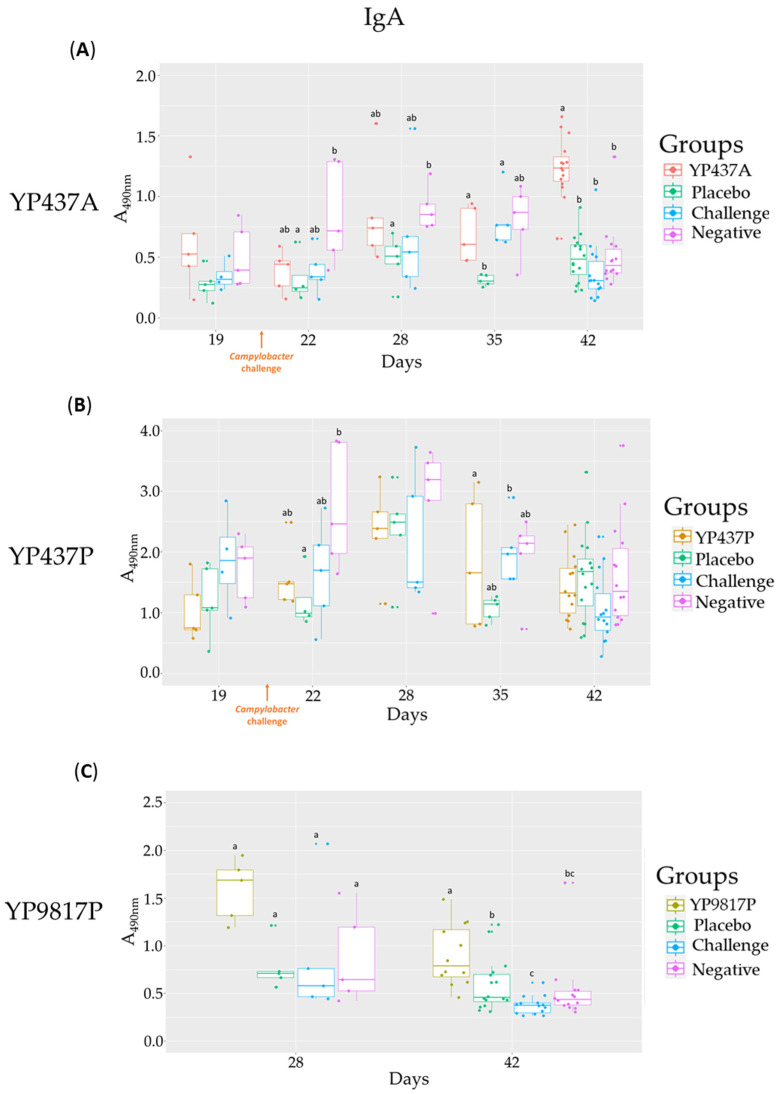
Specific anti-YP437A, anti-YP437P and anti-YP9817P IgA levels in bile from D19 to D42 in vaccinated groups and control groups. Each point represents one chicken. (**A**) Specific anti-YP437A IgA (**B**) Specific anti-YP437P IgA (**C**) Specific anti-YP9817P IgA. To compare antibody levels between the groups on each test day, an ANOVA parametric test was used when the normality and homogeneity criteria of the variances had been validated; otherwise, the non-parametric Kruskall-Wallis test was applied. Significant differences (*p* ≤ 0.05) between groups estimated using the Tukey test after an ANOVA parametric test or the Wilcoxon test after the Kruskall-Wallis test are represented by superscript letters ^abc^.

First, no significant difference in the levels of specific antibodies (IgY and IgA) against the three vaccine proteins was observed between the three control groups (placebo, challenge and negative) (Figure 3A–C and Figure 4A–C).

Moreover, the dilutions of proteins for coating and antibodies used for analysis of anti-YP9817P antibody levels (IgY and IgA) were higher than for YP437A, which were even higher than for YP437P (Table 1).

Furthermore, specific IgY antibodies were observed in the serum of all vaccinated chickens. The production of specific anti-YP9817P IgY on D28 and D42, anti-YP437A IgY from D22 to D42 and anti-YP437P IgY on D35 and D42 were significantly higher in the vaccinated groups than in the control groups (Figure 3A–C). In that respect, in chickens vaccinated with YP9817P and YP437A vaccines, the specific systemic immune response appeared earlier (D28) (Figure 3A,C) than with the YP437P vaccine (D35) (Figure 3B).

Moreover, a significantly higher production of specific IgA was measured in the bile of chickens vaccinated with YP437A and YP9817P on D42 compared with the control groups, except in chickens vaccinated with YP437P (Figure 4A–C). A higher inter-individual variability was observed in the results of the specific anti-YP437P IgA ELISA for all groups (vaccinated and controls) (Figure 4B).

Thus, there is a different specific humoral immune response depending on the vaccine and the production batch. For the vaccine, the specific humoral immune response in the YP9817P and YP437A groups occurred earlier than in the YP437P one. For the production batch, there was an earlier specific humoral immune response in the YP437A group than in the YP437P one.

### 3.4. Relative Gene Expressions Determined by RT-qPCR

Relative expressions of eight caecal cytokines and two β-defensin genes (that were previously shown to play a role in response to *Campylobacter* infection and vaccination against *Campylobacter*) were evaluated on caecal tissues on D42 by relative RT-qPCR. 

**Figure 5:**
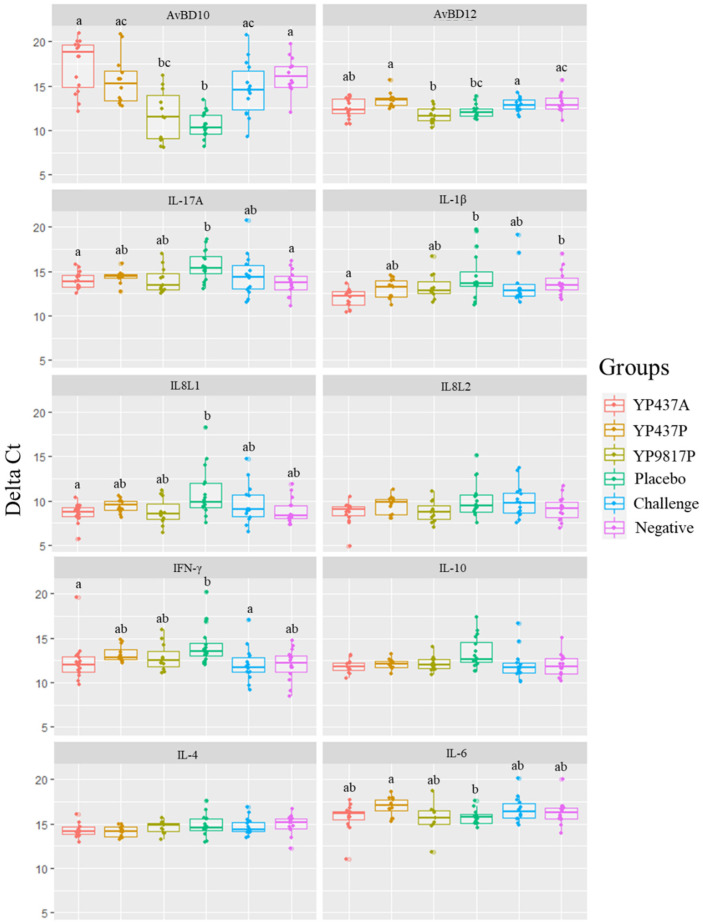
Expressions of cytokines and β-defensins in the caecum on D42. Each gene’s expression, represented by delta Ct, corresponds to the average of the differences in the duplicate Ct for each gene and the duplicate Ct for each of the two reference genes (GAPDH and β-actin). Each point represents one chicken. The higher values represent a lower expression while smaller values represent a higher expression. For each gene, statistical tests were performed using the mean of the duplicates of 2^-ΔCt^ data obtained thanks to the two reference genes. The non-parametric Kruskall-Wallis test was applied. Significant differences (*p* ≤ 0.05) between groups were estimated using the Wilcoxon test and are represented by superscript letters ^abc^. The genes evaluated were host defence peptide avian β-defensins 10 (AvBD10) and AvBD12, interleukin (IL)17-A, IL-1β, IL8L1, IL8L2, interferon (IFN)-γ, IL-10, IL-4, IL-6.

First, no significant difference in the expression of all the tested genes was observed between the challenge group and the negative group on D42, reflecting no effect of *Campylobacter* colonisation on both cytokine and β-defensin production (Figure 5). However, the placebo group demonstrated an upregulation of AvBD10 and AvBD12 (significantly higher delta Ct) and a downregulation of IFN-γ (significantly lower delta Ct) compared with the challenge group (*p* ≤ 0.05). Contrastingly, no difference (*p* > 0.05) was observed between the placebo group and the negative group concerning the relative expression of AvBD12 and IFN-γ (Figure 5).

No significant difference in relative gene expressions was observed between the YP9817P and the placebo groups. An upregulation of IL-17A, IL-1β, IL8L1, and IFN-γ (significantly higher delta Ct) and a downregulation of AvBD10 (significantly lower delta Ct) in the YP437A groups compared with the placebo (*p* ≤ 0.05) was observed, but not compared with the challenge and negative groups (*p* > 0.05). Similarly, the YP437P vaccine induced a downregulation of AvBD10, AvBD12, and IL-6 (significantly lower delta Ct) compared with the placebo group (*p* ≤ 0.05) but not compared with the challenge and negative groups (*p* > 0.05) (Figure 5). These discrepancies in the results observed between the vaccinated groups and the placebo group or with the other control groups (challenge and negative groups) did not allow a conclusion to be reached about the effect of the three vaccines on the expression of the tested genes.

### 3.5. Caecal Microbiota Analyses

To assess the impact of vaccination with YP437A and YP9817P candidates on caecal microbiota, the caecal content of the chickens in the different groups was analysed on D42 using 16S metabarcoding. The effect of the YP437P protein was not evaluated due to the low immune response demonstrated in this study. The number of sequences after read demultiplexing and pre-processing (merging, denoising and dereplication) was 3,253,573 (minimum: 22,614; maximum: 117,822).

A total of 713 OTUs were obtained (with 1,698,589 sequences). The minimum and maximum number of OTUs in each group is indicated in Table 4.

**Figure 6:**
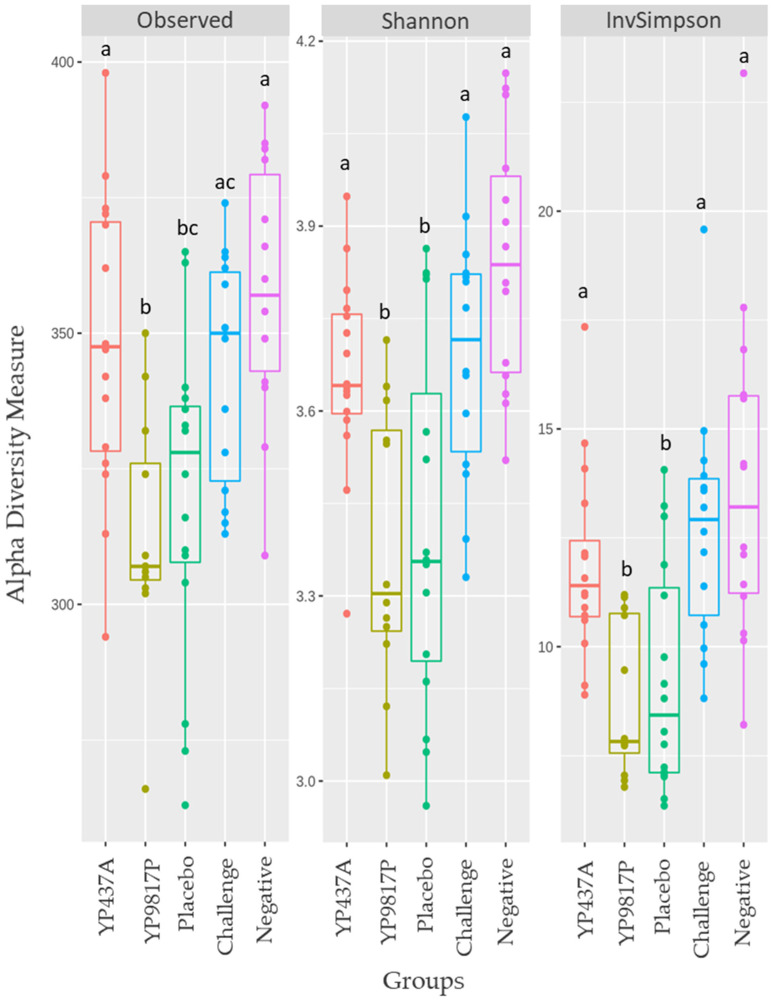
Richness and alpha diversity indices for the chickens’ caecal microbiota on D42. Each point represents one chicken. To compare the groups, the parametric ANOVA test was applied. Significant differences (*p* ≤ 0.05) between groups were estimated using the Tukey test and are represented by superscript letters ^abc^.

**Figure 7:**
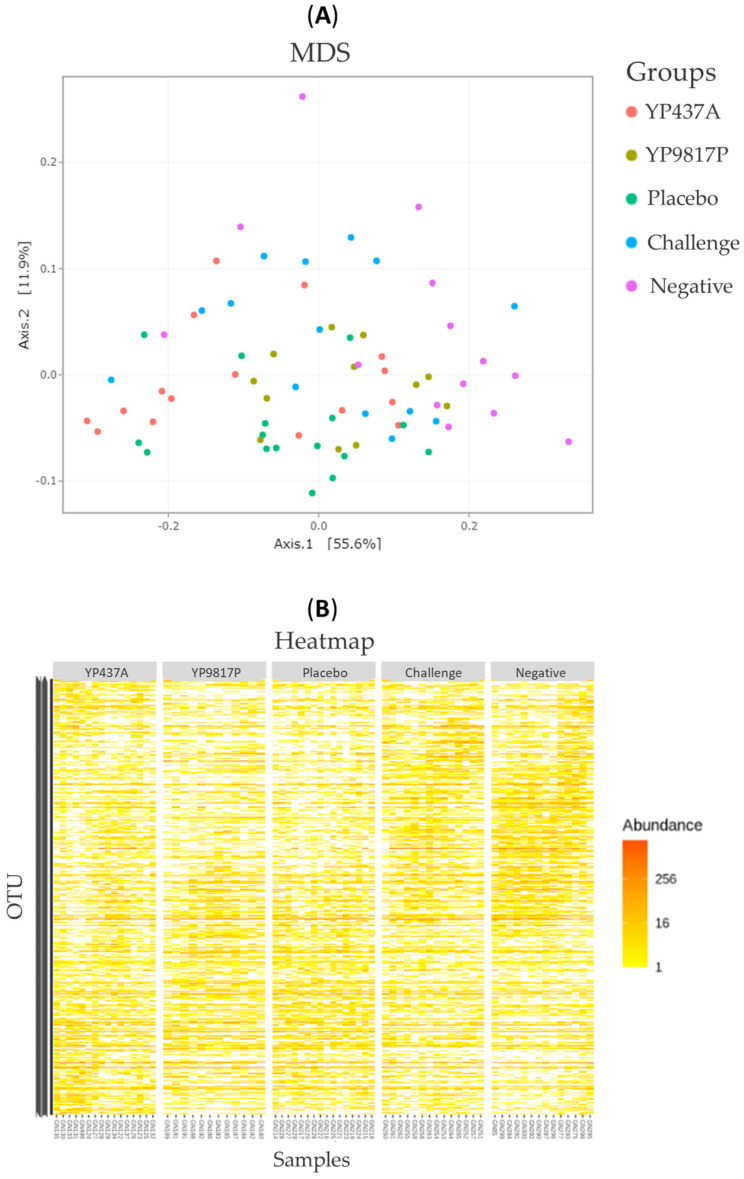
Representation of the beta diversity for the chickens’ caecal microbiota on D42. (**A**) MDS based on weighted UniFrac distance. Each point represents one chicken. (**B**) The heatmap represents the community structure. The relative abundance of OTUs is represented by the colour scale, yellow being the least and red the most abundant.

To determine bacterial diversity, the alpha and beta diversities of the caecal microbiota were assessed (Figure 6 and Figure 7). The richness (observed species) and diversity (Shannon and inverse Simpson indices) indices were compared between groups for the alpha diversity found within samples (Figure 6). Both richness and diversity were higher (*p* ≤ 0.05) in the YP437A, challenge, and negative groups than in the YP9817P and placebo groups (Figure 6). These results suggest that the YP437A, challenge and negative groups contained a larger number of species (higher richness) more evenly distributed than the YP9817P and placebo groups.

For the analysis of beta diversity, the differences in microbial population compositions among the YP437A, YP9817P, placebo, challenge, and negative groups were examined by multi-dimensional scaling (MDS) based on weighted UniFrac distance (Figure 7A). Little segregation of the groups was observed, suggesting that the most abundant OTUs in the different groups are phylogenetically close (Figure 7A). The heatmap, representing the community structure with the relative abundance of OTUs, also confirmed the minimal segregation of the groups observed (Figure 7B). However, a multivariate ANOVA (performed with Adonis) revealed that the bacterial community structure differed significantly (*p*  ≤  0.001) between groups and that the group explained 25% of the observed variation.

**Figure 8:**
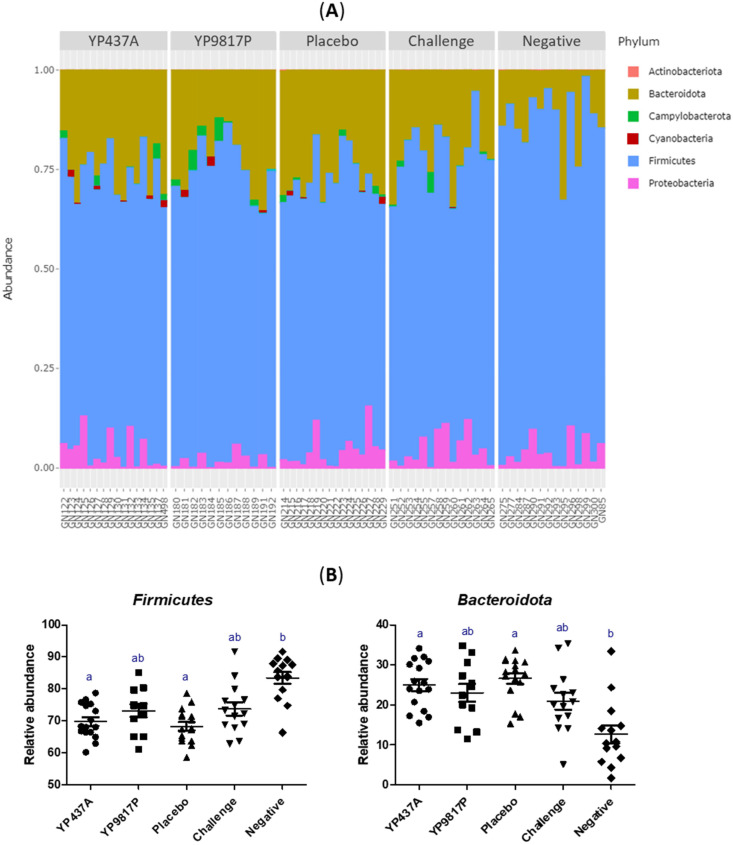
Composition of the caecal microbiota structure at the phylum level on D42. (**A**) Relative abundance of the six phyla identified in caecal microbiota. Each bar represents one sample. (**B**) Relative abundance of the two phyla which differed significantly between groups. Each point represents one chicken. To compare the groups, the non-parametric Kruskall-Wallis test was applied. Significant differences (*p* ≤ 0.05) between groups were estimated using the Wilcoxon test and are represented by superscript letters ^ab^.

The caecal bacterial composition in each group at the phylum level on D42 is represented in Figure 8. According to the results, the *Firmicutes* phylum predominated in each group, followed by *Bacteroidota* and *Proteobacteria* phyla (Figure 8A). Among the predominant phyla, the relative abundance of the *Firmicutes* phylum was significantly higher in the negative group than in the YP437A and placebo groups (*p* ≤ 0.05), but not in the YP9817P and challenge groups (*p* > 0.05). On the contrary, the *Bacteroidota* phylum was significantly lower in the negative group than in the YP437A and placebo groups (*p ≤* 0.05), but not in the YP9817P and challenge groups (*p* > 0.05) (Figure 8B). These results suggest that *Campylobacter* decreases the relative abundance of *Firmicutes* and increases the relative abundance of *Bacteroidota*, whereas the vaccination did not have any effect on the relative abundance of these phyla. The relative abundance of the *Proteobacteria* phylum did not differ significantly between the groups.

**Figure 9:**
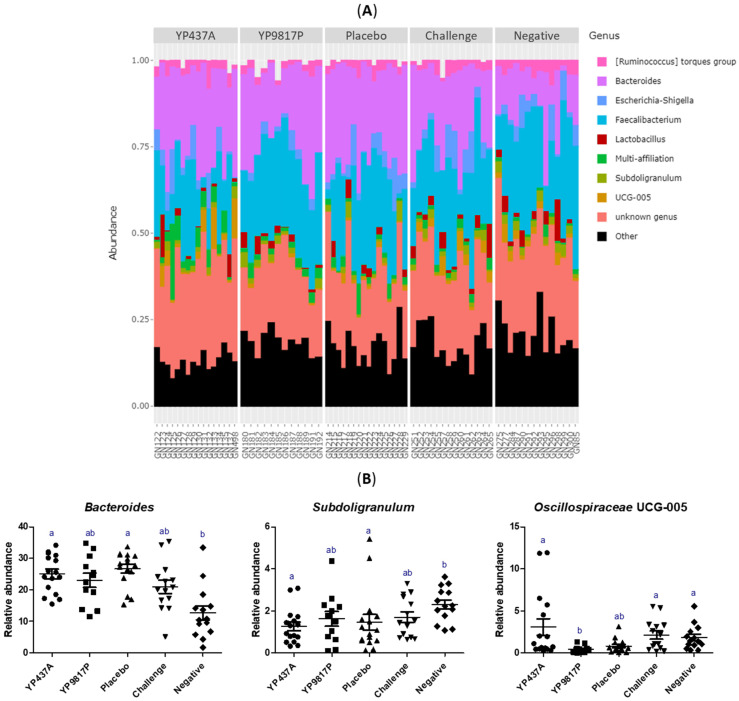
Composition of the caecal microbiota structure at the genus level. (**A**) Relative abundance of the nine main genera identified in caecal microbiota represented by a bar for each sample. (**B**) Relative abundance of the major genus with a significant difference between the groups. Each point represents one chicken. To compare the groups, the non-parametric Kruskall-Wallis test was applied. Significant differences (*p* ≤ 0.05) between groups were estimated using the Wilcoxon test and are represented by superscript letters ^ab^.

The caecal bacterial composition in each group at the genus level (nine main genera) on D42 is represented in Figure 9. The *Faecalibacterium* and *Bacteroides* genera predominated in each group, followed by *Escherichia-Shigella*, [*Ruminococcus*] torque group, *Lactobacillus*, UCG-005 (*Oscillospiraceae* family), and the *Subdoligranulum* genus (Figure 9A). Among the predominant genera, the relative abundance of the *Bacteroides* genus (*Bacteroidota* phylum) was significantly lower in the negative group than in the YP437A and placebo groups (*p* ≤ 0.05) but not lower than in the YP9817P and challenge groups (*p* > 0.05). On the contrary, the *Subdoligranulum* genus (*Firmicutes* phylum) was significantly higher in the negative group than in the YP437A and placebo groups (*p* ≤ 0.05) but not higher than in the YP9817P and challenge groups (*p* > 0.05) (Figure 9B). These results suggest that *Campylobacter* decreases the relative abundance of the *Subdoligranulum* genus (*Firmicutes* phylum) but increases the relative abundance of the *Bacteroides* genus (*Bacteroidota* phylum), which agrees with the analysis of relative abundance at the phylum level. Moreover, the relative abundance of *Oscillospiraceae* UCG-005 (*Firmicutes* phylum) was significantly lower in the YP9817P group than in the YP437A, challenge and negative groups (*p* ≤ 0.05) but not lower than in the placebo group (*p* > 0.05) (Figure 9B), reflecting a possible impact of the YP9817P vaccination on the relative abundance of Oscillospiraceae UCG-005. The relative abundance of the other predominant genera (*Faecalibacterium, Escherichia-Shigella*, [*Ruminococcus*] torque group and *Lactobacillus*) did not significantly differ between the groups.

In addition, to identify the characteristic bacteria which were specific to each group, a linear discriminant analysis (LDA) effect size (LEfSe) algorithm approach was applied. However, no specific taxa were identified for each group. These results indicate that no OTUs with a statistically different relative abundance between the groups were identified. Consequently, the effect of vaccination and *Campylobacter* colonisation are not taxon-specific.

## 4. Discussion

Vaccinating broilers against *Campylobacter* is one of the possible strategies to reduce *Campylobacter* caecal load and, consequently, reduce the risk of the incidence of human campylobacteriosis. Many vaccine research studies have been carried out in the past few decades, but the results have been inconclusive and inconsistent [45,46,47]. To this date, therefore, the mechanisms of protection against *Campylobacter* following vaccination remain to be fully elucidated, and currently vaccines against *Campylobacter* are not available. It is also necessary to have a vaccine platform that can help evaluate the immune and protective potential of newly identified potential vaccine antigens. DNA vaccination is one such platform because of the ease with which novel antigens may be included. A previous review reported DNA vaccination in poultry as an appropriate and simple strategy with promising results against bacteria (e.g., *Campylobacter jejuni*, *Chlamydophila psittaci*). Such vaccination can boost the protective immune response while avoiding maternal immunity [48]. It is to be noted that heterologous DNA prime/other recombinant vectors or recombinant proteins are often associated. In a previous study, we used reverse vaccinology to identify potential vaccine candidates against *Campylobacter* [38], and some of these vaccine candidates have already been tested using a DNA prime/protein boost vaccine regimen. These vaccines induced a partially protective immune response against *Campylobacter*, but the protections were not confirmed in a second trial [24]. It was speculated that the absence of significant protection in the second trial may have been due to high inter-individual *Campylobacter* colonisation rate differences (including the non-vaccinated group), thus masking a potential reduction in colonisation within some vaccine groups. One other hypothesis is that the protein vaccine batch affected vaccine efficacy in certain cases. Nevertheless, since these vaccine candidates induced partial protection once, they may be used again to evaluate several points. Firstly, to conclude regarding the protection against *Campylobacter* induced by antigens YP437 and YP9817 (i.e., two of the promising antigens) administered via the prime DNA/protein boost regimen, they were tested once again in this work, as was the potential effect of the vaccine production batch. Secondly, other parameters were evaluated to better characterise the immune responses and changes in microbiota induced after vaccination against *Campylobacter* colonisation; this will potentially help to identify avenues for improving the efficacy of future vaccines against *Campylobacter* in poultry.

In the present study, the colonisation of broilers by *Campylobacter* was evaluated from D22 to D42, but no protection was observed against *Campylobacter* with the three vaccines tested. Moreover, *Campylobacter* caecal loads presented a high inter-individual variability in all groups. These results have already been observed after a *Campylobacter* challenge in previous in vivo studies [24,49,50] and in broiler flocks [7].

In this work, a specific antibody response was tested in serum for the three tested antigens. A specific systemic immune response was observed for the YP437A vaccine (the same batch as previously used), confirming the immune potential of this antigen [24] and its quicker response than the YP437P vaccine (the new batch used only here), revealing that the batch can have an impact. Moreover, the YP9817P vaccine induced production of specific anti-YP9817P IgY in serum, also confirming the previous results [24], even though the batch was not the same. Despite the production of specific IgY in serum, no effect on *Campylobacter* colonisation was observed. As already suggested, IgY might have been absent or inaccessible (denatured, degraded, or both) during the passage through the intestine, thereby reaching the caeca at insufficient concentrations, or it might not have been active in the caeca [14,51,52].

A large majority of the IgA involved in mucosal immune responses is present in the bile [53]. In this study, significant quantities of specific IgA antibodies were produced in the YP9817P and YP437A groups (belatedly, on D42) but not in the YP437P group. The discrepancies between the two YP437 groups again indicate a batch effect on the induction of immune responses. More specifically, in the YP437A group, specific IgA appeared to increase over time without reducing *Campylobacter* colonisation. These results have already been observed in another study where there appeared to be an increase in IgA over time after the challenge by *Campylobacter* without, however, affecting *Campylobacter* colonisation [53]. On the other hand, the absence of a specific mucosal response observed in chickens in the YP437P group could have been due to the short half-life of IgA [54] or to the interference of maternal antibodies as has been previously demonstrated [25]. Maternal antibodies could block interactions between the circulating pathogen-derived antigens and immune cells [55].

The limited effect of humoral immunity (IgY and IgA production) on *Campylobacter* caecal load was already suggested by an immature bursa of Fabricius leading to an immature antibody repertoire of up to 5–7 weeks [56]. On the contrary, other studies have found that humoral immunity appears to be involved in the reduction of *Campylobacter* colonisation in birds [21,57] and, more specifically, in the mucosal immune response [58]. However, high antibody levels do not necessarily indicate in vivo protective power in broilers, as was previously observed when using these proteins [24] and other proteins of *Campylobacter* such as flagellin [17] or CjaA [59]. Antibodies need to bind protein epitopes, and it is essential that immunogenic conformational epitopes are available on the protein’s surface. As already suggested, the folding of a recombinant protein is not necessarily equivalent to the protein’s native structure and might expose epitopes normally hidden, thereby leading to a false-positive ELISA response [60].

It could be beneficial to determine the three-dimensional structure of the recombinant protein by X-ray crystallography to compare it with the three-dimensional structure model of the native protein in *Campylobacter*. However, in vivo assays—which are dependent on the protein of interest—represent the best probe for the native structure. Moreover, additional tests could be performed, such as determining N-terminal signal peptides or investigating the possibility of an alternative secretion pathway to obtain more information on the vaccinal potential of proteins. In fact, the N-terminal part could elicit a strong antibody response [60]. Thus, the combination of several conventional laboratory and bioinformatic tests could provide additional answers.

It is noteworthy that the production of specific antibodies (IgA and IgY) against the tested antigens was also observed in the negative group, whereas there was no *Campylobacter* in this group and therefore no specific antibody production was expected. These results could be explained by cross-reactions between the coated antigens and the presence of antibodies targeting similar protein domains on bacteria in the commensal microbiota.

Contrary to other studies from 2 days to 22 days post inoculation [32,36,37,61], we found no impact of *Campylobacter* on cytokine and β-defensin production on D42 (23 days post inoculation). Moreover, because the relative expression of certain genes was upregulated in vaccine groups compared with the placebo group but not when compared with the challenge and negative groups, it was not possible to reach a conclusion on the role of vaccination in innate and cellular response. We can also highlight that there was no significant difference in gene expression between the YP437A and YP437P vaccinated groups. Thus, there is no difference in innate and cellular responses depending on the production batch. However, one author found that cellular pathways appear to be stimulated by *Campylobacter* colonisation and not by vaccination until a certain level of tolerance to *Campylobacter* colonisation is reached [62]. Thus, the role played by these cytokines in the elaboration of a response remains to be explained.

In this study, the humoral immune responses were strongly stimulated in response to the three vaccines against *Campylobacter*, whereas the results regarding the expression of immunity genes were inconclusive. These results could be explained by the structure of epitopes. In fact, the epitopes exposed to the surface were probably conformational ones with discontinued residues. Moreover, it has been estimated that up to 90% of B cell epitopes from native proteins are conformational rather than linear ones [63].

The *Firmicutes*, *Bacteroidota* and *Proteobacteria* phyla predominated in the caecum of chickens, a situation consistent with previous reports [64,65,66,67]. The results of this study suggest that the presence of *Campylobacter* could be associated with a lower relative abundance of *Firmicutes* but a higher relative abundance of *Bacteroidota*. However, the contrary was observed in other studies [68,69]. Among the *Bacteroidota* phylum, *Campylobacter* seems to be associated with a higher relative abundance of the *Bacteroides* genus, but in another study, this genus was reported in flocks not colonised by *Campylobacter* [70]. *Campylobacter* seems to be associated with a lower relative abundance of the *Subdoligranulum* genus (*Firmicutes* phylum). In the same way, one study reported that *Subdoligranulum* occurred more often in *Campylobacter*-free flocks [70], whereas another study reported an increase in *Subdoligranulum* when exposed to *C. jejuni* [49]. The relative abundance of the *Oscillospiraceae* UCG-005 genus (*Firmicutes* phylum) was significantly lower in the YP9817P group than in the YP437A, challenge, and negative groups, suggesting that the type of vaccine could modulate the relative abundance of specific taxa in the caecal microbiota. Indeed, another study with a DNA prime/protein boost flagellin-based vaccine revealed an increase in the *Ruminococcaceae* and *Bacillaceae* families (*Firmicutes* phylum) in response to vaccination against *Campylobacter* [17]. Other studies on vaccination against *Campylobacter* have reported changes and in particular a high abundance of taxa belonging to the *Firmicutes* phylum [15,17,71]. However, another study has shown that the abundance and diversity of chicken gut microbiota were not impacted by three vaccinations with the Ent–KLH conjugate [18].

In this study, the DNA prime/protein boost strategy inoculated intramuscularly led, for all three vaccines, to strong systemic and mucosal antibody immune responses, but the implication of cytokines and β-defensins on D42 and major changes in the microbiota (measured only for vaccines YP437A and YP9817P) were not demonstrated. Moreover, the three vaccines did not offer protection against *Campylobacter* colonisation. A batch effect was observed between the YP437A and YP437P groups concerning the production of specific antibodies but not concerning the expression of cytokines and β-defensins, probably due to a different protein conformation or purity. Further investigations are needed, perhaps using another administration strategy by including other vectors or by evaluating other routes of inoculation such as the oral or in ovo routes. In fact, an important consideration in the vaccination strategy used in this study is that the antigens were delivered via intramuscular injection, resulting in a robust IgY antibody response. However, as *Campylobacter* is in the digestive tract of chickens, mucosal antibodies (e.g., mucosal delivery) may further reduce the colonisation of *C. jejuni*. A mucosal delivery of vaccine has previously demonstrated promising results against *Campylobacter* in the literature [22,29,72].

## 5. Conclusions

This study revealed that no reduction in *Campylobacter caecal* load was observed despite the induction of humoral and mucosal immune responses, particularly with YP437A and YP9817P vaccines. Moreover, a batch effect on the induction of immune responses was observed.

Further studies are needed to identify the role of immune responses and microbiota composition against *Campylobacter*. Future protocols of vaccination could be tested in broilers with other vaccine regimens (e.g., mucosal and in ovo).

## Figures and Tables

**Table 1:** Conditions of protein coating and antibody dilutions for each protein in serum and bile.

Protein	Coating (µg/mL)	Dilution of Primary Antibody	Dilution of Secondary Antibody
Serum			
YP437A	0.5	1:100	1:25,000
YP437P	2.0	1:50	1:25,000
YP9817P	0.2	1:100	1:35,000
Bile			
YP437A	0.5	1:100	1:5000
YP437P	2.0	1:50	1:2000
YP9817P	0.2	1:100	1:2000

**Table 2:** Primer sequences for the gene expression of IL-6 and GAPDH determined by qPCR.

Target Gene	Primer Sequence (5′-3′)	Product Size (bp)	NCBI Accession Number	Reference
IL-6	F: CGTGTGCGAGAACAGCATGGAGA R: TCAGGCATTTCTCCTCGTCGAAGC	110	NM_204628.1	[42]
GAPDH	F: TGACGTGCAGCAGGAACACT R: AATACGGCCAAATCCGTTGAC	67	NM_204305.1	Determined using Primer Express^®^

**Table 3:** Body weights (presented as mean ± SD in g) of chicken groups at different time points during the trial. To compare body weights between the different groups each day, an ANOVA parametric test was used when the normality and homogeneity criteria of the variances had been validated; otherwise, the non-parametric Kruskall-Wallis test was applied. Significant differences (*p* ≤ 0.05) between groups and the negative group were estimated using the Tukey test after an ANOVA parametric test or the Wilcoxon test after the Kruskall-Wallis test and are indicated by “(*p* ≤ 0.05)”.

Groups	Day 5	Day 12	Day 19	Day 22	Day 28	Day 35	Day 42
YP437A	113 ± 9	353 ± 30	776 ± 86	995 ± 116	1538 ± 252	2283 ± 373	3042 ± 517
YP437P	111 ± 9	354 ± 24	788 ± 73	1031 ± 106	1582 ± 169	2355 ± 265	3285 ± 255
YP9817P	115 ± 7	355 ± 23	763 ± 86	1091 ± 240	1606 ± 232	2296 ± 376	3060 ± 446
Placebo	114 ± 9	352 ± 31	767 ± 74 (*p* < 0.05)	1018 ± 109	1629 ± 203	2506 ± 375	3314 ± 509
Challenge	111 ± 8	377 ± 27	846 ± 72	1095 ± 92	1724 ± 174	2574 ± 274	3927 ± 484
Negative	109 ± 9	362 ± 36	812 ± 136	1069 ± 148	1610 ± 292	2462 ± 472	3483 ± 526

**Table 4:** Minimum and maximum OTUs per group.

Groups	OTUs (Min)	OTUs (Max)
YP437A	424	567
YP9817P	370	435
Placebo	343	484
Challenge	402	503
Negative	349	519

## Data Availability

Not applicable.

## Supplementary Materials

The following supporting information can be downloaded at: https://www.mdpi.com/article/10.3390/pharmaceutics15051397/s1, Table S1: Alpha diversity index values for all the chicken caecal samples on D42; Table S2: Table showing relative abundance of OTUs and their taxonomic affiliation in chicken caecal samples on day 42.
